# A multicenter, randomized, double-blind, placebo-controlled trial evaluating the efficacy and safety of Tong Luo Hua Shi capsule, a modernized Tibetan medicine, in patients with rheumatoid arthritis

**DOI:** 10.1186/s13063-016-1481-3

**Published:** 2016-07-27

**Authors:** Wei Liu, Yuan-Hao Wu, Si-Yuan Hu, Cheng-Liang Zhong, Ming-Li Gao, Dong-Wu Liu, Hai-Yun Wang, Mu-Zhi Chen, Yue-Jin Song, Ben-Zha-Xi Yang, Qing-Shan Zheng, Hua Yao, Xue-Bing Qi, Gang Li

**Affiliations:** 1 First Teaching Hospital of Tianjin University of Traditional Chinese Medicine, An-shan-xi Road #314, Nankai District, Tianjin 300193 China; 2 Affiliated Hospital of Liaoning University of Traditional Chinese Medicine, Shenyang, China; 3 Traditional Chinese Medicine Hospital of Xinjiang Uygur Autonomous Region, Urumqi, China; 4 Hubei Hospital of Traditional Chinese Medicine, Wuhan, China; 5 Tibetan Medicine Hospital of Qinghai Province, Xi’ning, China; 6 Center for Drug Clinical Research, Shanghai University of Traditional Chinese Medicine, Shanghai, China; 7 Lanzhou Heshengtang Pharmaceutical Co. Ltd., Lanzhou, China; 8 Beijing Highthink Pharmaceutical Service Technology Co. Ltd., Beijing, China

**Keywords:** Rheumatoid arthritis, Tibetan medicine, Antirheumatic therapy, Tong Luo Hua Shi capsules, Wu-wei-gan-lu decoction

## Abstract

**Background:**

Tong Luo Hua Shi (TLHS) is a new formulation of the traditional Tibetan medicine Wu-wei-gan-lu that has been used for the treatment of rheumatoid arthritis (RA) for hundreds of years in China. This study aimed to evaluate the efficacy and safety of TLHS in patients with RA.

**Methods:**

This was a randomized, double-blind, placebo-controlled, dose-finding study performed in patients with active RA from five medical centers. Patients received three doses (4.8, 3.6, or 2.4 g/day po) of TLHS or placebo (tid po) for 8 weeks. Blood sampling, physical examination, and assessment of the American College of Rheumatology (ACR) 20 % improvement (ACR20) criteria were performed before and every 2 weeks after starting treatment. The primary endpoint was the ACR20. The secondary endpoints included safety.

**Results:**

A total of 240 participants were screened and 236 patients were randomized (*n* = 59/group); 20 dropped out. After 8 weeks, ACR20 improvements in the TLHS 4.8 g and 3.6 g groups were significantly higher than in the placebo group (*P* < 0.01 and *P* < 0.05, respectively). ACR50 improvement in the TLHS 4.8 g group was significantly higher compared with the placebo group (*P* < 0.01). Symptoms of RA were significantly relieved in the TLHS groups. In the TLHS groups, insomnia (*n* = 1), gastroenteric reactions (*n* = 2), arrhythmia (*n* = 1), and minor hepatic lesion (*n* = 1) were reported; in the placebo group, hepatic dysfunction (*n* = 1) was reported (*P* = 0.878).

**Conclusions:**

TLHS improved the symptoms of patients with RA according to the ACR20. Moreover, TLHS was safe.

**Trial registration:**

Chinese Clinical Trial Registry: ChiCTR-TRC-12003871. Registered on 1 January 2012.

**Electronic supplementary material:**

The online version of this article (doi:10.1186/s13063-016-1481-3) contains supplementary material, which is available to authorized users.

## Background

Rheumatoid arthritis (RA) is a chronic inflammatory disease characterized by persistent synovitis and progressive destruction of cartilage and bone with the presence of rheumatoid factors. RA is also associated with systemic inflammatory manifestations in addition to local inflammation of multiple joints [1–3]. The prevalence of RA is 0.35 % in women and 0.13 % in men [4]. Risk factors include smoking, genetic factors, elevated rheumatoid factor levels, elevated soluble tumor necrosis factor receptor II levels, coffee consumption, absence of hormonal replacement therapy, and posttraumatic stress disorder [5–9]. Treatment of RA involves the control of inflammation using anti-inflammatory drugs [1, 2].

Tong Luo Hua Shi (TLHS) capsules are a new formulation of the traditional Wu-wei-gan-lu decoction, which is one of the basic medicines in traditional Tibetan medicine for the treatments of different diseases, especially RA, and has been used for hundreds of years in China [10–12]. TLHS is made from herbs including *Salvia miltiorrhiza*, *Ephedra intermedia*, *Sabina przewalskii*, *Myricaria paniculata*, *Artemisia sieversiana*, *Astragalus membranaceus*, and *Rhododendron anthopogonoides* [10–12]. Previous studies of the Wu-wei-gan-lu decoction revealed its efficacy for the treatment of acute gouty arthritis [13] and RA [14, 15].

Therefore, the aim of the present study was to evaluate the efficacy and safety of TLHS in patients with RA.

## Methods

### Study design

This was a multicenter, randomized, double-blind, placebo-controlled, dose-finding trial. It was reported according to the recommendation of the Consolidated Standards of Reporting Trials (CONSORT) [16]. The trial was approved by the State Food and Drug Administration of China (No. 2006 L03370) and the ethical committee of The First Teaching Hospital of Tianjin University of Traditional Chinese Medicine (No. TYLL2009015). Written informed consent was obtained from each participant before enrollment. The trial was registered with the Chinese Clinical Trial Registry (registration number: ChiCTR-TRC-12003871; date of registration: 1 January 2012). The manuscript was in accordance with the populated CONSORT checklist (see Additional file 1) and flow diagram (see Additional file 2).

This study took place in five centers: the First Teaching Hospital of Tianjin University of Traditional Chinese Medicine, the Affiliated Hospital of Liaoning University of Traditional Chinese Medicine, the Traditional Chinese Medicine Hospital of Xinjiang Uygur Autonomous Region, the Hubei Hospital of Traditional Chinese Medicine, and the Tibetan Medicine Hospital of Qinghai Province.

### Participants

Participants (*n* = 236) were recruited between May 2009 and June 2011. Participants had to have been diagnosed with RA according to the American College of Rheumatology (ACR) 1991 revised criteria [17]. All participants had to have an ACR functional class of I, II, or III, and be in radiographic stage I, II, or III.

Exclusion criteria were: (1) pregnant or lactating women, and women of child-bearing age who were not using an effective method of contraception; (2) severe disability; (3) history of serious allergic reactions; (4) any other concurrent rheumatic disease such as systemic lupus erythematosus, Sjogren’s syndrome, or severe osteoarthritis; (5) significant cardiac, hematologic, respiratory, neurological, endocrine, renal, hepatic, gastrointestinal, or psychotic disease; (6) active recurrent infection; (7) alcoholism or drug dependency; or (8) psychological disorder.

Nonsteroidal anti-inflammatory drugs (NSAIDs) were discontinued at least 30 days before participation. Participants receiving a stable dosing regimen of the same glucocorticoids, e.g., prednisolone (10 mg daily maximum), or disease-modifying antirheumatic drugs (DMARDs) prior to entering the study were allowed.

If severe pain or complications occurred in a patient, making it impossible to participate in the study, as confirmed by the investigator, the patient was removed from the study. Poor compliance to treatment or study protocol led to removal from the study. Otherwise, patients could drop out of the study if they wished to; the reasons for dropping out were recorded. If a patient dropped out because of poor treatment effect, this patient was analyzed as achieving no treatment efficacy.

### Study medication and administration

The TLHS and placebo capsules were provided by Lanzhou Heshengtang Pharmaceutical Co., Ltd. (Lanzhou, China). Placebos were supplied in the form of capsules matched for weight, shape, and color.

The patients were randomized to receive 4.8, 3.6, or 2.4 g/day (three pills/day) of TLHS po or placebo po. All patients consumed four capsules each time and the dose was adjusted using a combination of TLHS and placebo capsules. An independent statistician prepared sequential sealed envelopes based on a random number table generated using SAS 9.2 (SAS Institute, Cary, NC, USA). Randomization was implemented without blocks. The envelopes and the allocation sequence were managed by a statistician. When a patient was recruited, the treating physician phoned the independent statistician, who then opened the next envelope and phoned the pharmacist to state the allocation. Capsules were prepared by the pharmacist. Patients and physicians were blinded to grouping.

According to the pain status and for all patients, including the patients in the placebo group, 25 mg of diclofenac sodium enteric-coated tablets (Voltaren, Beijing Novartis Pharmaceutical Co., Ltd., Beijing, China) bid or tid, and/or 20 mg of leflunomide (Fujian Huitian Biological Pharmaceutical Co., Ltd., Fuzhou City, China) were allowed, if necessary, after the second week of treatment, according to the treating physician’s judgment. The treating physician was blind to the study treatment.

### Endpoints

The primary efficacy endpoint was the improvement in ACR20 [18, 19]. Secondary endpoints were rheumatoid factor (RF) levels, C-reactive protein (CRP) levels, erythrocyte sedimentation rate (ESR), ACR50, ACR70, and safety.

### Efficacy and safety

Safety was monitored until the last administration of TLHS or placebo. Frequency and severity of adverse effects and adverse drug reactions were observed at each visit. Clinical and laboratory tests such as tender and swollen joint counts based on Disease Activity Score 28 (DAS28) [20], morning stiffness, average grip strength of two hands, 100-mm visual analog scale (VAS), and Health Assessment Questionnaire (HAQ)-disability index, were assessed by the investigators at screening, baseline, and at weeks 2, 4, and 8. Standard hematological and biochemical tests and urine analysis were also performed at each study site.

### Statistical analysis

No data about TLHS was available in the literature on which to base the power analysis. However, based on data about Wu-wei-gan-lu, we estimated that the effective rate should be about 50 % in the control group and about 75–80 % in the 4.8 g group. By setting the power at 80 %, α at 0.05, and the same sample size in each group, the minimal sample size for each group was between 39 and 59. By considering the drop-out rate, limitation of funds, and probable errors in estimating the effective rate, we decided to include 60 patients in each group.

The efficacy analysis was performed using the intent-to-treat principle, which includes all randomized participants who underwent at least one post-treatment evaluation. The last observation carried forward method was used to substitute for missing data. The safety analysis was performed on all participants who received at least one dose of study medication.

Continuous data are presented as mean ± standard deviation (SD) and were analyzed using one-way analysis of variance (ANOVA) and Tukey’s post hoc test. Categorical data are presented as frequencies and were analyzed using the chi-square test or Fisher’s exact test, as appropriate. Statistical testing, unless otherwise stated, was two-sided and used a 5 % significance threshold. Data were analyzed with SAS 9.2 (SAS Institute, Cary, NC, USA).

## Results

### Characteristics of the participants

**Fig. 1:**
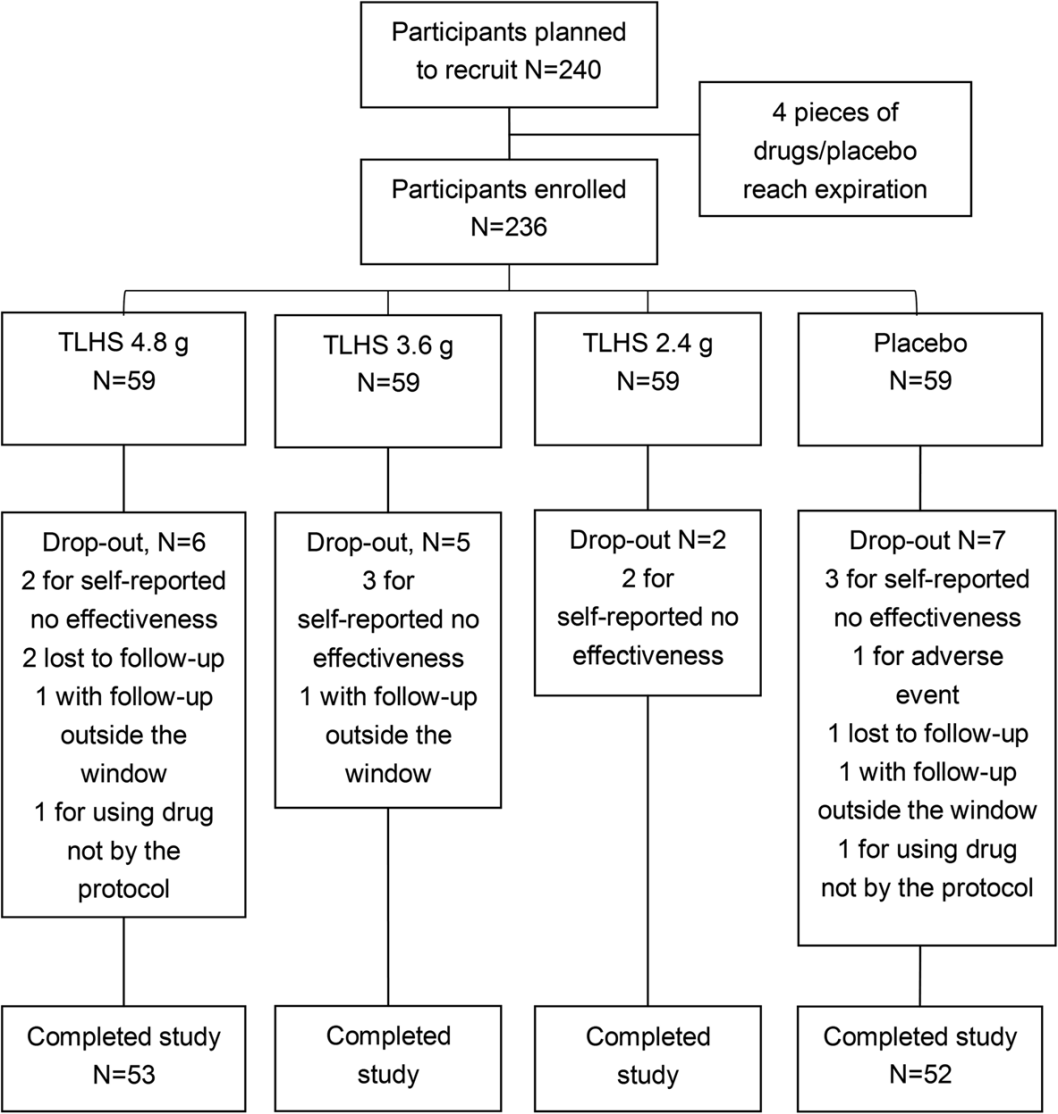
Flowchart of the participants

A total of 240 participants from five centers were screened between May 2009 and June 2011 and provided a written informed consent (Fig. 1); 216, 236, and 236 participants were included in the per-protocol set (PPS), the full analysis set (FAS), and the safety set (SS), respectively. Four patients could not be randomized because four sets of drugs/placebo (one in each group) had reached the expiration date before recruiting a subject. The decision was then taken to stop the study because the scientific committee wanted to use the same batch of drugs for all patients. Twenty participants dropped out (Fig. 1).

Except for the radiographic stage (*P* = 0.031), there was no clinically significant difference between the groups at baseline (all *P* > 0.10) (Table 1).

**Table 1:** Baseline characteristics of the participants

Characteristic	TLHS 4.8 g	TLHS 3.6 g	TLHS 2.4 g	Placebo
Age, years, mean ± SD	47.54 ± 11.14	50.76 ± 9.69	50.59 ± 10.61	50.12 ± 10.45
Gender, *n*, M : F	12:47	11:48	8:51	7:52
Height, cm, mean ± SD	162.88 ± 6.49	162.08 ± 6.16	161.78 ± 5.40	162.37 ± 5.60
Weight, kg, mean ± SD	61.83 ± 9.87	61.04 ± 9.65	60.14 ± 9.04	61.88 ± 9.29
Marital status, married : other	53:6	56:3	55:4	56:3
Duration of disease, months	3.00–180.00	2.00–216.00	5.00–183.00	2.00–276.00
Medication, use : no use	20:39	28:31	32:27	28:31
Radiographic stage, I : II : III	30:25:4	23:32:4	27:22:10*	33:21:5
Joint functional stage, I : II : III	13:46:0	9:47:3	9:42:8	15:40:4
Pain (VAS), cm, mean ± SD	5.68 ± 1.12	5.68 ± 1.27	5.48 ± 1.19	5.52 ± 1.13
Tender joint counts, *n*, mean ± SD	9.12 ± 5.07	8.47 ± 5.06	7.36 ± 3.87	9.24 ± 5.33
Swollen joint counts, *n*, mean ± SD	6.27 ± 3.47	5.83 ± 3.34	5.20 ± 2.54	6.19 ± 3.57
Morning stiffness, min, mean ± SD	82.03 ± 37.73	81.36 ± 37.61	83.31 ± 50.88	90.25 ± 71.24
Grip strength, mmHg, mean ± SD	36.11 ± 25.84	32.53 ± 22.57	35.07 ± 24.00	34.87 ± 24.97
Physician’s assessments, score, mean ± SD	5.32 ± 1.12	5.34 ± 1.33	5.07 ± 1.11	5.30 ± 1.17
HAQ, score, mean ± SD	16.68 ± 11.87	18.10 ± 12.78	15.39 ± 11.09	17.07 ± 12.85
RF, U/ml, mean ± SD	163.69 ± 258.04	74.72 ± 110.88	99.19 ± 149.96	79.04 ± 131.09
CRP, mg/dl, mean ± SD	7.38 ± 15.13	11.11 ± 30.01	6.83 ± 12.42	7.49 ± 16.67
ESR, mm/h, mean ± SD	22.39 ± 19.73	27.21 ± 25.14	27.93 ± 25.00	26.39 ± 23.04

*P < 0.05 versus the placebo group

*VAS* visual analog scale, *HAQ* Health Assessment Questionnaire, *RF* rheumatoid factor, *CRP* C-reactive protein, *ESR* erythrocyte sedimentation rate

### Safety

Treatment tolerance was good. In the TLHS 4.8 g group, one case of insomnia and one case of gastroenteric reaction were reported. In the 3.6 g group, there was one case of arrhythmia. In the 2.4 g group, one case of upset stomach and one case of minor hepatic lesion were reported. A case of hepatic dysfunction was reported in the placebo group. There was no significant difference in the incidences of adverse events (*P* = 0.963) or adverse drug reactions (*P* = 0.878) between the groups (Table 2). No serious adverse event occurred in the trial.

**Table 2:** Adverse events and adverse drug reactions

Event/reaction	TLHS 4.8 g	TLHS 3.6 g	TLHS 2.4 g	Placebo
Adverse events, *n* (%)	3 (5.1)	2 (3.4)	3 (5.1)	3 (5.1)
Adverse drug reactions, *n* (%)	2 (3.4)	1 (1.7)	2 (3.4)	1 (1.7)

### Effective rate of the ACR improvement

After 8 weeks, the improvement rates of the ACR20 in the TLHS 4.8 g and 3.6 g groups were significantly higher than in the placebo group (*P* = 0.012 and *P* = 0.035 in FAS, respectively; *P* = 0.002 and *P* = 0.016 in PPS, respectively). The improvement rate of the ACR20 in the 4.8 g group was higher than in the TLHS 2.4 g group (*P* = 0.020 in FAS; *P* = 0.004 in PPS). The improvement rate of the ACR50 in the TLHS 4.8 g group was significantly higher compared with the placebo group (*P* = 0.001 in FAS; *P* = 0.002 in PPS) and the TLHS 2.4 g group (*P* = 0.002 in FAS; *P* = 0.001 in PPS) (Table 3).

**Table 3:** Effective rate according to the ACR20, ACR50, and ACR70

	FAS, *n* (%)			PPS, *n* (%)		
	ACR70	ACR50	ACR20	ACR70	ACR50	ACR20
TLHS 4.8 g	6 (10.2)	24 (40.7)**^,^****	45 (76.3)*^,^***	6 (11.3)	23 (43.4)**^,^****	44 (83.0)**^,^****
TLHS 3.6 g	2 (3.4)	14 (23.7)	43 (72.9)*	2 (3.7)	14 (25.9)	42 (77.8)*^,^***
TLHS 2.4 g	1 (1.7)	9 (15.3)	33 (55.9)	1 (1.8)	9 (15.8)	33 (57.9)
Placebo	3 (5.1)	8 (13.6)	32 (54.2)	3 (5.8)	8 (15.4)	29 (55.8)

*ACR* American College of Rheumatology response criteria, *FAS* full analysis set, *PPS* per protocol set

*P < 0.05 versus the placebo group

**P < 0.01 versus the placebo group

***P < 0.05 versus the 2.4 g group

****P < 0.01 versus the 2.4 g group

### Secondary outcomes

The symptoms of the participants after treatments were significantly relieved. There were significant differences in the symptoms of RA between the TLHS groups and the placebo group after 8 weeks of treatments (Table 4).

**Table 4:** Condition of the participants at end of the trial

Indications	FAS				PPS			
	TLHS 4.8 g	TLHS 3.6 g	TLHS 2.4 g	Placebo	TLHS 4.8 g	TLHS 3.6 g	TLHS 2.4 g	Placebo
Pain (VAS), cm, mean ± SD	2.62 ± 1.37**	3.31 ± 1.30**	3.64 ± 1.29	3.99 ± 1.42	2.48 ± 1.30**	3.24 ± 1.32*	3.62 ± 1.30	3.89 ± 1.45
Pain changes, cm, mean ± SD	−3.06 ± 1.95**	−2.37 ± 1.31**	−1.85 ± 1.51	−1.53 ± 1.70	−3.31 ± 1.87**	−2.44 ± 1.25*	−1.88 ± 1.51	−1.63 ± 1.74
Tender joint counts, *n*, mean ± SD	3.88 ± 3.73**	4.80 ± 3.24	4.24 ± 2.54*	5.63 ± 4.10	3.96 ± 3.89	4.80 ± 3.38	4.19 ± 2.53	5.67 ± 4.32
Tender joint changes, *n*, mean ± SD	−5.24 ± 3.54**	−3.68 ± 3.19	−3.12 ± 2.88	−3.61 ± 3.51	−5.66 ± 3.46	−3.81 ± 3.24	−3.21 ± 2.88	−3.96 ± 3.54
Swollen joint counts, *n*, mean ± SD	2.24 ± 2.09**	2.68 ± 1.82	3.03 ± 1.88	3.41 ± 2.49	2.30 ± 2.13	2.56 ± 1.77	3.02 ± 1.90	3.31 ± 2.47
Swollen joint changes, *n*, mean ± SD	−4.03 ± 3.58*	−3.15 ± 2.48	−2.17 ± 2.43	−2.78 ± 2.96	−4.38 ± 3.61*	−3.31 ± 2.43	−2.30 ± 2.37	−3.04 ± 3.01
Morning stiffness, min, mean ± SD	36.53 ± 24.69**	46.10 ± 26.57	50.14 ± 36.08	54.93 ± 41.78	35.00 ± 23.27**	45.56 ± 27.29	50.14 ± 36.67	55.21 ± 44.38
Morning stiffness changes, min, mean ± SD	−45.51 ± 34.71	−35.25 ± 21.40	−33.17 ± 28.42	−35.32 ± 42.23	−50.09 ± 33.56	−38.15 ± 19.96	−33.81 ± 28.70	−39.12 ± 43.33
Grip strength, mmHg, mean ± SD	47.68 ± 28.59	38.80 ± 24.49	40.58 ± 26.27	38.84 ± 25.12	47.14 ± 29.13	36.00 ± 23.63	39.63 ± 26.22	36.99 ± 25.07
Grip strength changes, mmHg, mean ± SD	11.57 ± 21.19**	6.27 ± 7.07	5.51 ± 10.01	3.97 ± 8.53	12.32 ± 22.21**	6.76 ± 6.98	5.70 ± 10.13	3.93 ± 8.75
Physician’s assessments, score, mean ± SD	2.65 ± 1.40**	3.32 ± 1.37	3.62 ± 1.34	3.79 ± 1.42	2.52 ± 1.34**	3.24 ± 1.37	3.62 ± 1.36	3.68 ± 1.45
Physician’s assessments changes, score, mean ± SD	−2.67 ± 1.91**	−2.02 ± 1.36	−1.45 ± 1.48	−1.51 ± 1.65	−2.92 ± 1.84**	−2.07 ± 1.30	−1.46 ± 1.49	−1.60 ± 1.71
HAQ, score, mean ± SD	10.31 ± 9.91	12.69 ± 11.05	11.36 ± 9.64	12.92 ± 11.35	11.19 ± 10.06	12.89 ± 11.25	11.65 ± 9.67	13.60 ± 11.64
HAQ changes, score, mean ± SD	−6.37 ± 8.60	−5.41 ± 6.98	−4.03 ± 5.60	−4.15 ± 7.90	−6.89 ± 8.89	−5.83 ± 7.13	−4.18 ± 5.64	−4.31 ± 8.13
RF, U/ml, mean ± SD	122.61 ± 194.75	60.55 ± 99.04	90.35 ± 160.68	64.88 ± 102.79	129.88 ± 203.21	63.42 ± 104.21	93.28 ± 165.28	67.93 ± 109.24
CRP, mg/dl, median ± IQR	12.63 ± 44.50	5.68 ± 10.62	5.48 ± 8.48	6.81 ± 15.15	14.05 ± 47.27	5.75 ± 11.03	5.78 ± 8.70	7.25 ± 16.20
ESR, mm/h, mean ± SD	21.24 ± 21.91	21.32 ± 24.15	22.95 ± 20.24	23.25 ± 19.66	20.69 ± 21.89	21.39 ± 25.25	23.75 ± 20.42	24.26 ± 20.56
Joint functional stage, I : II : III	22:37:0	14:44:1	16:40:3	20:37:2	21:32:0	14:39:1	16:38:3	19:32:1
Voltaren use : no use	3:56**	10:47**	20:38**	23:33	3:50**	9:45**	20:37	21:31
Frequency of Voltaren use, mean ± SD	7.40 ± 8.08**	17.80 ± 13.02**	22.03 ± 17.11**	39.32 ± 20.13	7.40 ± 8.08**	17.63 ± 13.35**	22.03 ± 17.11**	39.29 ± 20.34
Leflunomide use : no use	14:45	22:35	22:36	24:32	41:39	20:34	22:35	22:30

*FAS* full analysis set, *PPS* per protocol set, *VAS* visual analog scale, *HAQ* Health Assessment Questionnaire, *RF* rheumatoid factor, *CRP* C-reactive protein, *ESR* erythrocyte sedimentation rate

*P < 0.05 and **P < 0.01 versus the placebo group. There were 226 patients in these four groups. Ten patients dropped out before the first follow-up at 2 weeks. The other dropouts were included in this table using the last follow-up data as the final data

## Discussion

TLHS is a new formulation of the traditional Tibetan medicine Wu-wei-gan-lu decoction that has been used for the treatment of RA for hundreds of years in China. This study aimed to evaluate the efficacy and safety of TLHS in patients with RA. Results showed that after 8 weeks, ACR20 improvement in the TLHS 4.8 g and 3.6 g groups was significantly higher than in the placebo group. ACR50 improvement in the TLHS 4.8 g group was significantly higher compared with the placebo group. There was no difference in adverse events between the groups. TLHS improved the symptoms of patients with RA according to the ACR20. Moreover, TLHS was safe.

The quality of TLHS in China is controlled by HPLC analysis [21, 22]. In previous pharmacodynamics studies, TLHS revealed obvious inhibitory effects on swollen feet induced by albumen, formaldehyde, or adjuvant injections, and on edema of the ears of mice induced by xylene [23, 24]. In addition, studies have shown that TLHS reduced the writhing responses to acetic acid injection and tail flick against thermal stimulation [10, 25–29]. It can also increase the coagulation time and regulate the phagocytic immunity of macrophages in mice. Toxicology studies suggested that after giving high, moderate, or low doses of TLHS for 6 months, the hematologic, biochemical, and histopathological indexes were normal [10, 25–29]. Furthermore, experimental data showed that the maximum tolerable dose of TLHS was as high as 75.6 g/kg, which is equivalent to 300 times the clinical dose for adults. Previous clinical trials showed that the basic formula of TLHS, Wu-wei-gan-lu decoction, has immunoregulatory and antiarthritic effects for treating RA [14, 15].

The present study suggests that TLHS may be used to treat the symptoms of RA and to improve the quality of life, and that it is safe. On one hand, it demonstrated a good mid-term safety profile and good treatment compliance, in spite of some mild and transient adverse events or reactions including insomnia, gastroenteric reactions, and arrhythmias. On the other hand, the symptoms such as pain, tenderness, swelling, and morning stiffness were improved by TLHS. The patients’ grip strength and their conditions assessed by the physician could also be improved. Finally, fewer painkillers were needed by the patients. Nevertheless, it must be stressed that these results were obtained in a Chinese population and that traditional Chinese medicine is seldom used in Western countries.

The present study is not without limitations. Even if it was a multicenter trial, the sample size was relatively small. Second, only a limited panel of inflammatory markers was assessed. Further study is necessary to determine the comprehensive mechanisms of TLHS on inflammation. Larger studies are necessary to adequately demonstrate the effects of TLHS on RA. Other chronic inflammatory conditions might also benefit from TLHS.

## Conclusions

The results of the present study showed that TLHS is safe and effective in improving the primary effective values and reducing the secondary symptoms in patients with RA.

## Abbreviations

ACR, American College of Rheumatology; DMARD, disease-modifying antirheumatic drug; FAS, full analysis set; HAQ, Health Assessment Questionnaire; NSAID, Nonsteroidal anti-inflammatory drug; PPS, per-protocol set; RA, rheumatoid arthritis; RF, rheumatoid factor; SS, safety set; TLHS, Tong Luo Hua Shi; VAS, visual analog scale

## Additional files

Additional file 1: CONSORT 2010 Checklist. (DOC 217 kb) Additional file 2: CONSORT flow diagram. (PDF 483 kb)
