# Supplementary figures and images for: A multicenter, randomized, double-blind, placebo-controlled trial evaluating the efficacy and safety of Tong Luo Hua Shi capsule, a modernized Tibetan medicine, in patients with rheumatoid arthritis
Source: Trials. 2016 Jul 27;17:359. doi: 10.1186/s13063-016-1481-3 (PMC4963949; doi:10.1186/s13063-016-1481-3)

## CONSORT 2010 Flow Diagram

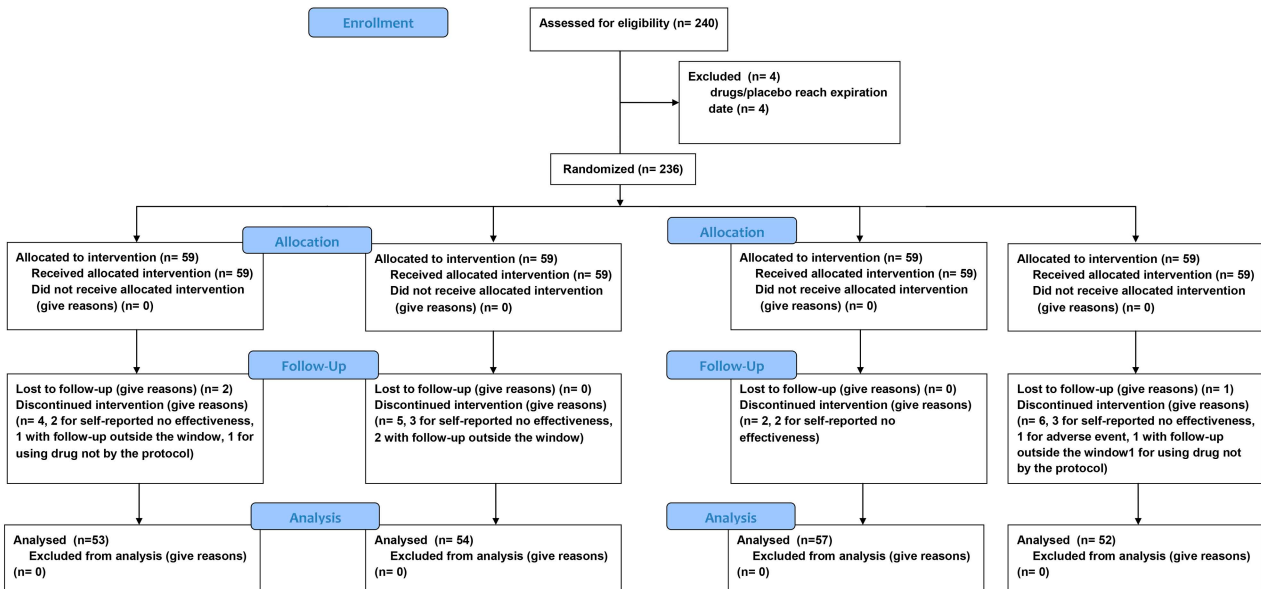

Supplement: Additional file 2: — CONSORT flow diagram. (PDF 483 kb) [file 13063_2016_1481_MOESM2_ESM.pdf]
